# Supplementary material for: The hidden side of animal cognition research: Scientists’ attitudes toward bias, replicability and scientific practice
Source: PLoS One. 2021 Aug 31;16(8):e0256607. doi: 10.1371/journal.pone.0256607 (PMC8407565; doi:10.1371/journal.pone.0256607)

Supporting Information

Our sample reported publishing most often in Animal Cognition (64.8% of the sample), Journal of Comparative Psychology (46.2%), Animal Behavior and Cognition (32.9%) and Frontiers in Psychology: Comparative Psychology (31.9%). A smaller proportion of our sample had published in Journal of Experimental Psychology: Animal Learning and Cognition (21.4%), and International Journal of Comparative Psychology (20.5%). Researchers most often endorsed the terms 'animal behavior' (78.5%) and 'animal cognition' (77.9%) as describing their research very or extremely well, followed by 'comparative psychology' (61.2%) and 'animal learning' (56.6%). A minority of researchers endorsed the terms 'behavioural ecology' (26.8%) and behavioural neuroscience (19.5%). Fig S1 shows these responses. An exploratory k-means clustering analysis found two well-fitting sets of clusters in the data. The first had two clusters. The first cluster was large (N = 144), and contained researchers endorsing the terms 'animal behaviour', 'animal cognition', 'animal learning' and 'comparative psychology', but not the terms 'behavioural ecology' and 'behavioural neuroscience'. The second cluster was smaller (N = 57), and contained researchers endorsing 'animal behaviour' and 'behavioural ecology' most strongly. This suggests our sample might have contained many general animal cognition researchers, and a smaller number of behavioural ecologists. An alternative pattern of clustering had four clusters. In addition to a cluster similar to the previous behavioural ecology cluster (N = 36), the general animal cognition researchers could be divided into those endorsing behavioural neuroscience (N = 54), avoiding behavioural neuroscience (N = 47), and those endorsing only animal behaviour, cognition, learning and comparative psychology (N = 64). Irrespective of the exact clusters, our sample appears to consist of animal researchers at all stages in their careers with a range of interests, largely reflecting the 6 journals we sampled.

S1 Fig: Researcher's opinions on how well a range of terms (animal behaviour, animal cognition, comparative psychology, animal learning, behavioural ecology and behavioural neuroscience) describes their research, $N = 210$. Percentages may not add to 100% due to a small number of NA responses not being visualised.

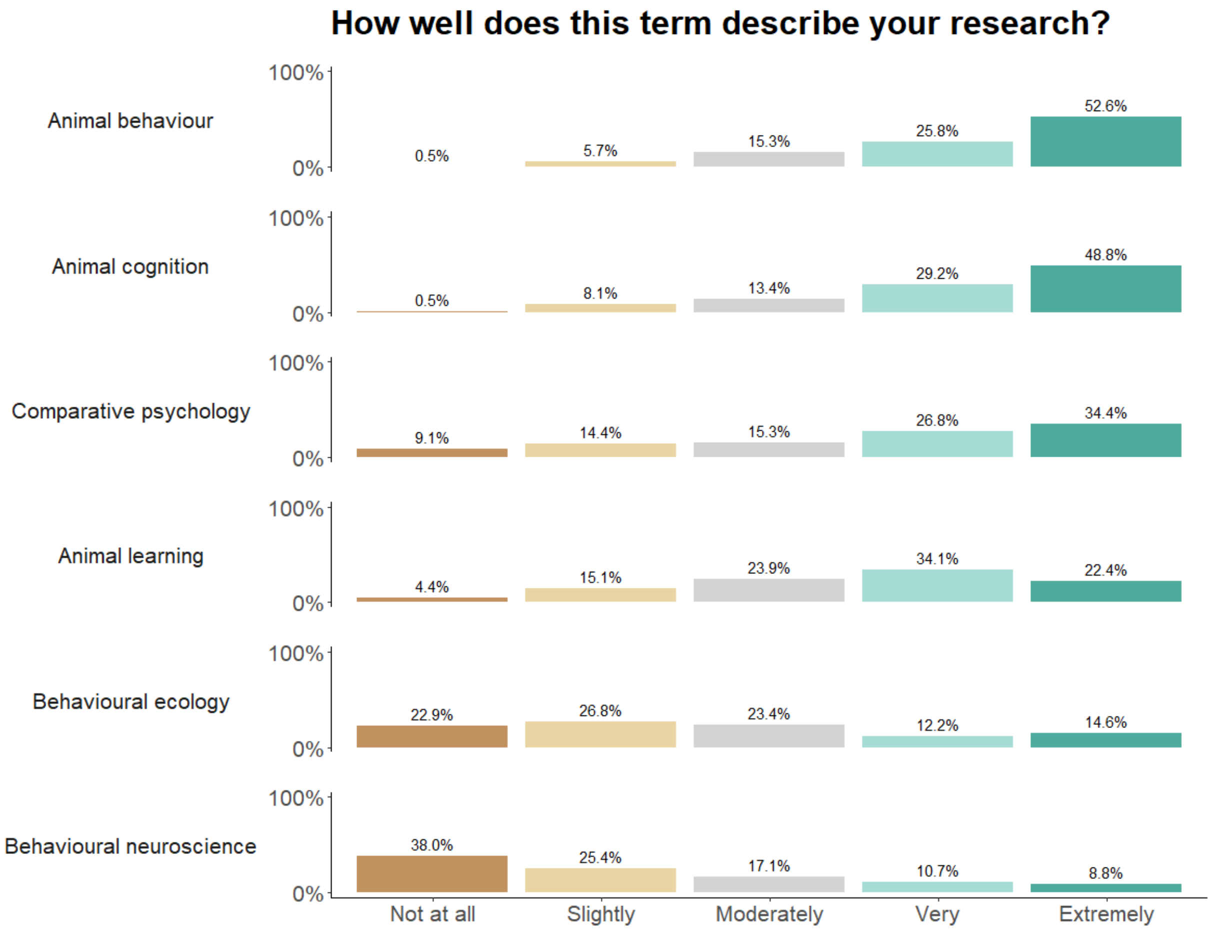

Supplement: S1 File — (PDF) [file pone.0256607.s001.pdf]
